# Effects of dietary chromium propionate and space allowance on performance and carcass responses of growing-finishing pigs

**DOI:** 10.1093/tas/txab112

**Published:** 2021-06-27

**Authors:** Alexandre P Santos, Mike D Tokach, Charles Kiefer, Robert D Goodband, Jason C Woodworth, Joel M DeRouchey, Steve S Dritz, Jordan T Gebhardt

**Affiliations:** 1 Animal Science Graduate Program, Federal University of Mato Grosso do Sul, Campo Grande, MS, Brazil; 2 Department of Animal Sciences and Industry, College of Agriculture, Kansas State University, Manhattan, KS 66506, USA; 3 Department of Diagnostic Medicine/Pathobiology College of Veterinary Medicine, Kansas State University, Manhattan, KS 66506, USA; 4 Genus PIC, Hendersonville, TN 37075, USA

**Keywords:** chromium propionate, finishing pig, space allowance

## Abstract

In a 72-d trial, 256 pigs (Line 600 × 241, DNA Columbus, NE) were used to determine the effect of dietary Cr (Cr propionate; Kemin Industries, Des Moines, IA) and physical space restriction on performance and carcass characteristics of finishing pigs. Pens were blocked by initial weight and randomly assigned to treatments with eight pigs per pen and eight pens per treatment. Treatments were arranged in a 2 × 2 factorial with main effects of Cr (control and Cr propionate, 200 µg/kg added Cr) and space allowances (0.91 m^2^/animal: normal and 0.63 m^2^/animal: restricted). Pigs were fed in three dietary phases and pigs were weighed approximately every 14 d throughout the study. Feed efficiency was calculated as both a standard gain to feed ratio and as an adjusted G:F ratio at a common final bodyweight. There were no evidence of space allocation × Cr interactions for any measured responses (*P* > 0.05). Space restriction decreased (*P* < 0.001) daily weight gain, final body weight, hot carcass weight, and daily feed intake, but increased carcass yield (*P* = 0.009) and decreased backfat depth (*P* = 0.003). Feed efficiency was greater for pigs provided a normal space allowance when adjusted for a common final bodyweight (*P* = 0.021), although no evidence of a difference was observed for unadjusted G:F (*P* = 0.687). Adding Cr to the diet reduced G:F on both an adjusted and unadjusted basis (*P* ≤ 0.021). There was marginally significant evidence that pigs provided Cr had lower average daily gain (*P* = 0.079) and final bodyweight (*P* = 0.056) compared to pigs not provided added Cr. There was marginally significant evidence that Cr resulted in greater backfat depth (*P* = 0.069), although no evidence of a difference in other carcass parameters were observed (*P* > 0.10). These results demonstrated that there were no interactions between Cr propionate and space allocation, illustrating that under the conditions of this study Cr propionate did not provide an advantage in growth performance or carcass characteristics in either adequate or restricted space allocation.

## INTRODUCTION

Pigs are subjected to several stressors in the production system. Of the most common stressors, physical space restriction is an issue in the finishing phase. Space restriction can impair animal performance (Thomas et al., 2017; Lei et al., 2018), and increase the incidence of abnormal behavior, including aggression, and tail biting (Anil et al., 2007). The mechanism by which performance is impaired when physical space is reduced is not well understood. However, changes in stress-related hormones (e.g., cortisol, ACTH), cytokines (e.g., TNF-α, IL-6), and behavioral responses may be the likely causes of decreased performance (Oh et al., 2010; Kim et al., 2017). There is evidence that in situations of stress, Cr supplementation in the diet has positive effects on animal production, as observed in cattle (Burton, 1995), broilers (Uyanik et al., 2002; Norain et al., 2013), laying Japanese quail (Sahin et al., 2002), and laying hens (Mirfendereski and Jahanian, 2015). There is also evidence that Cr supplementation improves immune status in pigs (Wang et al., 2007). Thus, it has been hypothesized that Cr supplementation can minimize the response to stressors (Ward et al., 1997; Kim et al., 2009; Liu et al., 2017), and improve performance and characteristics in finisher pig carcasses (Park et al., 2009; Wang et al., 2014).

However, the results of Cr supplementation on pig performance have been inconsistent. Several reports have indicated a positive effect of Cr by increasing weight gain, feed intake, increasing lean meat deposition, and decreasing fat deposition (Sales and Jancík, 2011; Li et al., 2013; Untea et al., 2017). Others, however, have reported less consistent responses (Tian et al., 2014; Wang et al., 2014; Tian et al., 2015; Marcolla et al., 2017; Gebhardt et al., 2019a; b). A number of reasons such as the source and concentration of Cr in the diet, time of supplementation, nutritional and health status, stress and immune status of the herd, life stage, and genetics may be involved in the variation of response in general performance to chromium supplementation observed in pigs. Our hypothesis was that there would be an interaction between Cr addition and space restriction in that the response to Cr supplementation might be greater in pigs stressed due to space restriction. In this context, this study was conducted with the objective of evaluating the effects of Cr (Cr propionate) supplementation on the performance and carcass characteristics of finishing pigs under physical space restriction as a form of stress.

## MATERIAL AND METHODS

### General

The Kansas State University Institutional Animal Care and Use Committee approved the protocol used in this experiment. The study was conducted at the Swine Teaching and Research Center at Kansas State University in Manhattan, KS. The facility was an environmentally regulated solid-sided building with completely slatted flooring. The study was conducted from March to June 2016, and barn temperature setpoint began at approximately 23 °C and was incrementally dropped to approximately 20 °C by the end of the study.

### Animals and Diets

A total of 256 pigs [128 castrated males and 128 females, line 600 × 241 DNA, Columbus, NE; initially 58.3 ± 0.65 kg bodyweight (BW) ± standard error of mean] were used over an experimental period of 72 d. Males were castrated at approximately 5 to 7 d of age using scalpel excision. The pens of animals were randomized to dietary treatment arranged in a 2 × 2 factorial with the main effects of diet (control and 200 µg/kg of added Cr propionate) and physical space (0.91 m^2^/animal: normal; and 0.63 m^2^/animal: restricted). Average pen BW served as a blocking factor and there were eight pigs per pen (four castrated males and four females) and eight pens per dietary treatment.

The pens were equipped with adjustable gates to allow for different space allowances per animal. The space per animal for each treatment was considered based on the prediction equations of Gonyou et al. (2006), and the *k* values ​​estimated for the animals’ weight range during the experimental period were 0.0353 and 0.0247 for animals in normal and restricted space, respectively. In the event of the removal or death of any animal in the stall during the experiment, the size of the stall was readjusted to maintain the same physical space as the treatment. Each stall was equipped with a feeder (Farmweld, Teutopolis, IL) with two 35.5 × 11.4 cm (length × width) feeding openings and a ladle-type drinking fountain.

The pens were located on a completely slatted concrete floor with a 120 cm pit for the storage of waste. A robotic feeding system (FeedPro; Feed logic Corp., Wilmar, MN) was used to supply and record the daily feed supply for each pen. The pigs received food and water ad libitum. The feed was manufactured at the O.H. Kruse Feed Technology Innovation Center, Manhattan, KS. The diets were composed of corn and soybean meal (Table 1), formulated for three phases. Ingredient nutrient profiles and standardized ileal digestibility coefficients were obtained from the NRC (2012), and diets were formulated to meet or exceed the NRC (2012) nutrient requirement estimates. Chromium propionate (KemTRACE Cr 0.04%; Kemin Industries, Des Moines, IA; 400 mg/kg total Cr) was added at a concentration of 0.45 kg/ton, replacing corn in the control diets for each phase, to provide 200 µg/kg of added Cr. Diets did not contain feed grade antimicrobials or ractopamine.

**Table 1.** Ingredient composition and calculated nutrient content of experimental diets (as fed-basis)^*a*^

Ingredient, %	Phase 1	Phase 2	Phase 3
Corn	77.85	80.55	83.85
Soybean meal, 46% CP	19.70	17.25	13.95
Limestone	1.00	1.00	1.00
Monocalcium phosphate, 21% P	0.40	0.30	0.30
Salt	0.35	0.35	0.35
L-Lys	0.30	0.25	0.23
L-Thr	0.08	0.06	0.06
DL-Met	0.06	0.02	0.01
L-Trp	0.02	0.01	0.01
Trace mineral premix^*b*^	0.10	0.10	0.10
Vitamin premix^*c*^	0.10	0.10	0.10
Phytase^*d*^	0.02	0.02	0.02
Cr propionate^*e*^	+/-	+/-	+/-
Total	100	100	100
Calculated analysis, %			
Standardized ileal digestible (SID) amino acids			
Lys	0.90	0.80	0.70
Ile:Lys	62	64	65
Leu:Lys	142	153	164
Met:Lys	32	30	31
Met and Cys:Lys	58	58	61
Thr:Lys	62	63	66
Trp:Lys	19	19	19
Ca	0.51	0.48	0.47
P	0.43	0.40	0.38
STTD P	0.31	0.28	0.27
Metabolizable energy, Mcal/kg	3.31	3.32	3.32

^*a*^Phase 1 diets were fed from days 0 to 20 (58- to 75-kg BW); phase 2 experimental diets were fed from d 21 to 49 (75- to 105-kg BW); and phase 3 experimental diets were fed from d 50 to 72 (105- to 127-kg BW).

^*b*^rovided per kilogram of premix: 11 g Cu from copper sulfate, 0.2 g I from Ca iodate, 73 g Fe from ferrous sulfate, 22 g Mn from manganese sulfate, 0.2 g Se from sodium selenite, and 73 g Zn from zinc sulfate.

^*c*^Provided per kilogram of premix: 3,527,399 IU Vitamin A, 881,850 IU vitamin D3, 17,637 IU vitamin E, 15 mg vitamin B12, 3,307 mg riboflavin, 33,069 mg niacin, 11,023 mg pantothenic acid, and 1,764 mg menidione.

^*d*^Ronozyme Hiphos 2700 (DSM Nutritional Products, Inc., Parsippany, NJ), providing 540 phytase units (FYT)/kg and an estimated release of 0.09% STTD P. Estimated release of Ca was not included in calculated analysis.

^*e*^ 0.45 kg/ton of Cr propionate (Kemin Industries, Des Moines, IA) included as a substitute for corn in control diets to provide 200 µg/kg of added Cr.

### Data Collection

Pigs and feeders were weighed on d 0, 14, 28, 42, 56, and 72 to calculate daily feed intake, daily weight gain, and feed conversion. For animals removed from the study due to health reasons, the weight of the removed animal and days accrued within the period by the animal were included in the calculation of ADG, ADFI, and G:F. Dietary phase changes were made on d 20 and 49. An adjusted gain-to-feed ratio was calculated to consider the final body weight using the following equation:


$$G:F_{adj}=(124-LW_{72})\times 0.005+curent\;G:F$$

where 124 kg is the average of the final body weight with space restrictions, LW_72_ is the live weight at 72 d, 0.005 represents the slope estimate as described by Gaines et al. (2012), and current G:F is the unadjusted G:F.

On d 72, all pigs were individually weighed and tattooed with a unique identification number. Pigs were transported approximately 2.5 h to a commercial processing facility (Triumph Foods LLC, St. Joseph, MO) and held in lairage for approximately 7 h before slaughter. At the plant, hot carcass weight was taken immediately after evisceration with head removed and feet still attached to carcass. Backfat and loin depth were measured with an optical probe (Fat-O-Meter, SFK, Herlev, Denmark) inserted between the third and fourth rib (counting from the caudal end of the carcass) at a distance approximately 7 cm from the dorsal midline. Percentage lean was provided by the processing plant using proprietary equations. Carcass yield was calculated by dividing individual hot carcass weight obtained at the packing plant by the individual final live weight obtained at the farm.

### Statistical Analysis

Data were analyzed as a randomized complete block design using PROC GLIMMIX in SAS version 9.4 (SAS Institute, Inc., Cary, NC) with dietary treatment as a fixed effect and weight block as a random effect. The experimental unit was the pen for all analyses. Hot carcass weight was used as a covariate for analysis of backfat depth, loin depth, and percentage lean. Response criteria were tested for the main effects and potential interactions between Cr and space restriction. A Tukey multiple comparison adjustment was used when appropriate to control type I error rate. Average daily gain data and ADFI were also evaluated for each period throughout the study using the fixed effects of Cr, space, period, and all associated interactions. Block and the cross product of block, Cr, and space (representing pen) were included in the model as random effects and data were analyzed as repeated measures using a compound symmetry covariance structure. Significance was set at *P* < 0.05, and *P*-values falling within *P* > 0.05 and *P* < 0.10 were considered marginally significant.

## RESULTS

The results obtained show no evidence of an interaction between Cr and space restriction for the measured outcomes (*P* > 0.05; Table 2). There was marginally significant evidence for a space allocation × Cr interaction (*P* = 0.054), where pigs fed Cr when restricted in space allowance had slightly lower carcass yield compared to control-fed pigs, and pigs with adequate space had slightly greater carcass yield when Cr was provided compared to control. However, when evaluating using a Tukey multiple comparison adjustment, there was no evidence of a difference in carcass yield between Cr treatment and control within either space allocation (*P* > 0.05).

**Table 2.** Interactive effects of dietary Cr and space allocation on performance of growing-finishing pigs^*a*^

Space allocation:		0.91 m^2^		0.63 m^2^		SEM	*P*		
Item:	Diet:	Control	Cr	Control	Cr		Space × Cr	Space	Cr
BW, kg									
Day 0		58.3	58.3	58.3	58.3	0.65	1.000	0.940	0.881
Day 72		131.8	130.5	125.2	123.3	0.88	0.649	<0.001	0.056
Overall (d 0 to 72)									
ADG, kg		1.01	1.00	0.93	0.90	0.010	0.410	<0.001	0.079
ADFI, kg		2.93	2.96	2.68	2.66	0.027	0.385	<0.001	0.991
G:F		0.345	0.339	0.346	0.340	0.0031	0.939	0.687	0.021
G:F_adj_^*b*^		0.355	0.347	0.348	0.338	0.0034	0.877	0.021	0.015
Carcass characteristics^*c*^									
HCW, kg		96.9	96.7	93.1	91.3	0.71	0.231	<0.001	0.126
Carcass yield, %		72.08	72.38	72.69	72.48	0.149	0.054	0.009	0.729
Backfat depth, mm		22.05	23.39	20.14	20.53	0.584	0.301	0.003	0.069
Loin depth, mm		55.44	56.26	55.31	54.52	0.987	0.301	0.441	0.983
Lean, %^*d*^		50.92	50.55	51.54	51.25	0.284	0.849	0.060	0.131

^*a*^ A total of 256 pigs (Line 600 × 241, DNA Columbus, NE) were used in a 72-d trial growing-finishing trial with eight pigs per pen and eight pens per treatment. Pigs were housed with 0.91 m^2^ or 0.63 m^2^, and were provided either 0 or 200 µg/kg of added Cr propionate (Kemin Industries Inc., Des Moines, IA). A total of six pigs were removed from the trial due to health reasons (1 from 0.91 m^2^, control diet treatment; 2 from 0.91 m^2^, Cr diet treatment; 2 from 0.63 m^2^, control diet treatment; and 1 from 0.63 m^2^, Cr diet treatment).

^*b*^ Adjusted gain-to-feed ratio was calculated to adjust to a common final bodyweight.

^*c*^ Carcass characteristics other than yield were adjusted to a common HCW by inclusion of HCW as a covariate in the statistical model.

^*d*^ Percentage lean was provided by the processing plant using proprietary equations.

Pigs housed at 0.63 m^2^ had reduced (*P* < 0.001) feed consumption and daily weight gain in comparison to pigs housed at 0.91 m^2^ (Table 3). Feed conversion was not influenced by space restriction (*P* = 0.687), except when feed conversion was adjusted to a common body weight of 124 kg for all treatments. After adjusting for the difference in final weight, pigs under space restriction had decreased (*P* = 0.021) G:F than pigs at 0.91 m^2^.

**Table 3.** Main effects of dietary Cr and space allocation on performance of growing-finishing pigs^*a*^

	Space allocation, m^2^				Diet			
Item:	0.91	0.63	SEM	*P*	Control	Cr	SEM	*P*
BW, kg								
Day 0	58.3	58.3	0.65	0.940	58.3	58.3	0.65	0.881
Day 72	131.2	124.3	0.68	< 0.001	128.5	126.9	0.68	0.056
Overall (d 0 to 72)								
ADG, kg	1.01	0.92	0.007	< 0.001	0.97	0.95	0.007	0.079
ADFI, kg	2.94	2.67	0.019	< 0.001	2.81	2.81	0.019	0.991
G:F	0.342	0.343	0.002	0.687	0.346	0.339	0.002	0.021
G:F_adj_^*b*^	0.351	0.343	0.003	0.021	0.351	0.343	0.003	0.015
Carcass characteristics^*c*^								
HCW, kg	96.8	92.2	0.54	< 0.001	95.0	94.0	0.54	0.126
Carcass yield, %	72.23	72.58	0.121	0.009	72.38	72.43	0.121	0.729
Backfat depth, mm	22.72	20.34	0.420	0.003	21.10	21.96	0.324	0.069
Loin depth, mm	55.85	54.92	0.702	0.441	55.37	55.39	0.538	0.983
Lean, %^*d*^	50.73	51.40	0.210	0.060	51.23	50.90	0.164	0.131

^*a*^ A total of 256 pigs (Line 600 × 241, DNA Columbus, NE) were used in a 72-d trial growing-finishing trial with eight pigs per pen and eight pens per treatment. Pigs were housed with 0.91 m^2^ or 0.63 m^2^, and were provided either 0 or 200 µg/kg of added Cr propionate (Kemin Industries Inc., Des Moines, IA).

^*b*^ Adjusted gain-to-feed ratio was calculated to adjust to a common final bodyweight.

^*c*^ Carcass characteristics other than yield were adjusted to a common HCW by inclusion of HCW as a covariate in the statistical model.

^*d*^ Percentage lean was provided by the processing plant using proprietary equations.

**Figure 1.**
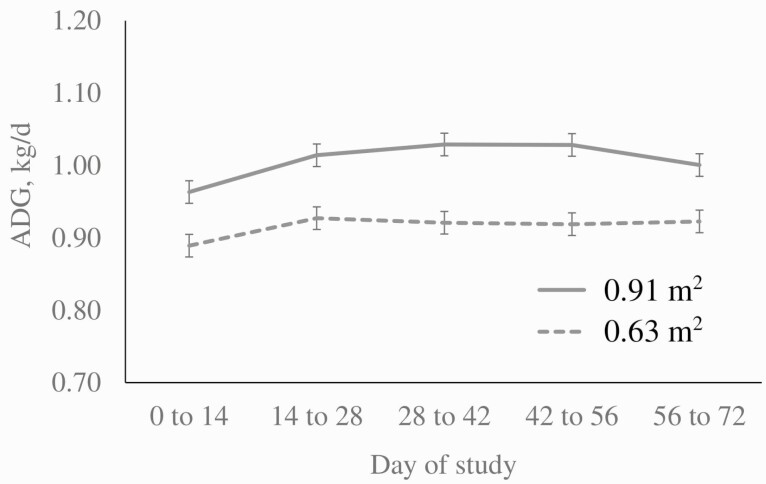
Average daily gain of pigs provided either 0.91 m^2^ or 0.63 m^2^ by period (space, *P* < 0.0001; period, *P* = 0.010; diet, *P* = 0.084; all interactions, *P* > 0.10; error bars represent ± SEM).

**Figure 2.**
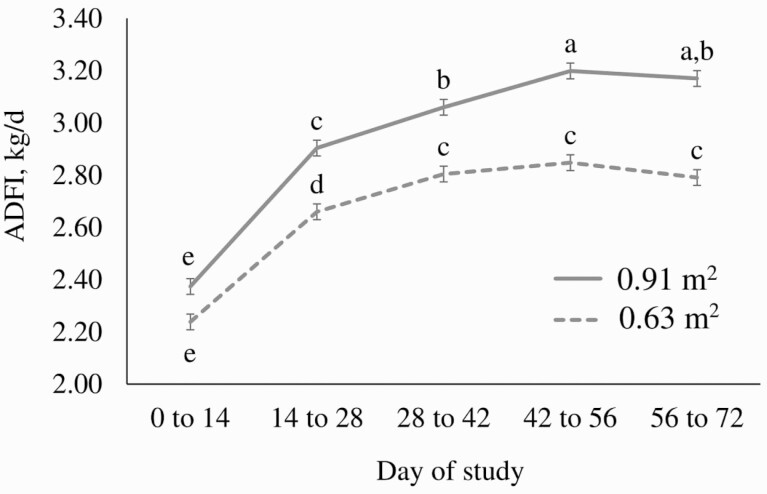
Average daily feed intake of pigs provided either 0.91 m^2^ or 0.63 m^2^ by period (space × diet × period, *P* = 0.058; space × period, space, and period, *P* < 0.0001; diet × period, diet × space, and diet, *P* > 0.10; Error bars represent ± SEM). Means lacking common superscript differ, *P* < 0.05, using a Tukey-Kramer multiple comparison adjustment.

Space restriction led to a reduction in average daily gain by period (*P* < 0.0001, Figure 1, largely driven by a reduction in ADFI (Figure 2). There was no evidence of a space × Cr × period or space × period interaction for ADG over the course of the study (*P* > 0.10). The body weights of pigs in the group housed with an area of ​​0.91 m^2^ were 1.41% (*P* = 0.013), 2.95% (*P* < 0.001), 4.04% (*P* < 0.001), 4.86% (*P* < 0.001), and 5.26% (*P* < 0.001) higher on d 14, 28, 42, 56, and 72, respectively, in comparison to animals housed at 0.63 m^2^. Cumulatively, the physical space restriction caused a reduction of 6.9 kg in the final weight of the animals (*P* < 0.001).

For carcass characteristics, pigs housed with an area of ​​0.63 m^2^ had lower (*P* < 0.001) hot carcass weight and backfat depth (*P* = 0.003), but had greater (*P* = 0.009) carcass yield. Furthermore, there was marginally significant evidence that pigs housed with an area of 0.63 m^2^ had greater percentage lean (*P* = 0.060). There was no evidence of a difference in loin depth between space allocations (*P* = 0.441).

Inclusion of Cr in the diet resulted in lower G:F (both adjusted and unadjusted, *P* ≤ 0.021). There was marginally significant evidence that inclusion of Cr in the diet resulted in lower ADG (*P* = 0.079) and there was a marginally significant difference in final BW (*P* = 0.056). Pigs fed Cr also had marginally greater backfat depth (*P* = 0.069), although there were no further differences in carcass characteristics attributed to Cr (*P* > 0.10). A total of 6 pigs were removed from the trial due to health reasons (1 from 0.91 m^2^, control diet treatment; 2 from 0.91 m^2^, Cr diet treatment; 2 from 0.63 m^2^, control diet treatment; and 1 from 0.63 m^2^, Cr diet treatment). There were no additional differences in growth performance or carcass characteristics attributed to Cr inclusion.

## DISCUSSION

Pigs are subjected to several stressors in the production system including space restriction during the finishing phase. It has been documented by numerous studies that reduced floor space results in reduced growth performance (Flohr et al., 2016; Johnston et al., 2017; Thomas et al., 2017; Carpenter et al., 2018; Lei et al., 2018; Wastell et al., 2018; Lerner et al., 2020) and increase the incidence of abnormal behavior, including aggression, and tail biting (Anil et al., 2007; Montoro et al., 2021). However, the negative outcome of increased prevalence of skin lesions in low space allowance conditions has not been observed in all studies as shown by Johnston et al. (2017). Therefore, it has clearly been established that stocking density can have a substantial impact on growth rate and several studies had demonstrated impacts on other important welfare measures.

The results of Cr supplementation on pig performance have been inconsistent in previously published literature. In the current study, it is not clear why inclusion of Cr reduced G:F and resulted in a marginally significant reduction in ADG. A meta-analysis performed by Sales and Jancík (2011) indicated that Cr supplementation would be associated with reduced backfat thickness and increased ADG, G:F, carcass lean, and loin muscle area. When further evaluating recently published literature, several studies have reported a positive effect of Cr by increasing weight gain, feed intake, increasing lean meat deposition, and decreasing fat deposition (Li et al., 2013; Untea et al., 2017). Others, however, have reported less consistent responses (Tian et al., 2014; Wang et al., 2014; Tian et al., 2015; Marcolla et al., 2017; Gebhardt et al., 2019a; b). Additional research is needed to further understand the response to dietary Cr when provided to growing and finishing pigs.

It has been hypothesized that Cr supplementation can minimize the response to stressors including restricted pen space (Ward et al., 1997) and heat stress (Kim et al., 2009; Liu et al., 2017; Mayorga et al., 2019). Therefore, the objective of the current experiment was the evaluate the impact of Cr supplementation in combination with chronic stress due to space restriction. Ward et al. (1997) evaluated the potential interaction of pen space, dietary crude protein level, and chromium tripicolinate addition in growing and finishing pigs and observed no meaningful interaction of space allowance and dietary Cr. In the current experiment, there were no evidence of space × Cr interactions which would be consistent with the previously reported work by Ward et al.

The use of the *k*-value proved to be a good reference point for predicting the ideal physical space per animal (Gonyou et al., 2006). The calculations in this study are similar to the expected loss of performance for restricted pigs and normal pigs (as applied in the industry for the finishing phase). In the current study, the reduction in space allowance per animal from 0.91 to 0.63 m^2^ impaired the performance of finishing pigs. Using a broken stick regression to identify the critical *k* value at which performance starts to decrease due to space constraints, Street and Gonyou (2008) reported a break point of *k* = 0.036 and Gonyou et al. (2006) reported a *k* of 0.034. Using these *k* values ​​as a reference, for the weight range of the animals in this experiment, the *k* value for the first 14 d of testing was 0.0365 for animals housed at 0.63 m^2^/animal, which is the threshold value of the break point reported by Street and Gonyou (2008) and Gonyou et al. (2006). The growth rate for pigs raised under space restriction had already been reduced during the first 14 d, despite the expectation that this growth restriction would not occur due to space in this experimental period. For all other periods evaluated, the *k* values ​​were all below 0.036, with values ​​reaching 0.0246 when the animals were between 111 and 127 kg. Pigs housed at 0.91 m^2^/animal, on the other hand, only reached *k* = 0.0352 at the end of the last period. Additionally, Flohr et al. (2018) recently updated prediction equations for ADG, ADFI, and G:F based on stocking density and other predictor variables. In the updated equations, *k* remains an important predictor of growth performance.

The hypothesis as to why the physical space restriction causes such a drastic effect on the general performance and pig carcass traits can be explained by the concept of allostasis (Goymann and Wingfield, 2004). In the present study, in which the animals were subjected to space restriction from day one, chronic stress occurs and the ripple effect of the restriction induces what is called an allostatic load, which is a new homeostatic adjustment point, resulting from the accumulation of allostatic responses that are chronic, excessive, or poorly regulated (Lee et al., 2015). To put this into context, consider a short-term space constraint that causes an increase in cortisol levels and, therefore, can be considered an allostatic response. This, in turn, triggers allostatic mediators, such as metabolic hormones, to act until the situation normalizes. If this event is recurrent (or intermittent) in space-restricted pigs, then chronic repetitive stress causes an allostatic overload, where cortisol and ACTH levels are generally higher than in animals with an adequate space allowance, and this becomes the new threshold for animals with restricted space.

Elevated levels of cortisol and ACTH, as well as other stress-related compounds, have been observed in animals under chronic stress conditions (Abbott et al., 2003; Li et al., 2017), and even though this was not tested in this study, this would be a convincing argument for why space-restricted pigs showed a statistically reduced performance during the experiment. Johnston et al. (2017) did not observe any difference in salivary cortisol concentrations based on floor space allowance when ranging from 0.71 to 1.07 m^2^/pig from 27 to approximately 140 kg BW. This approach could help to correlate physiological changes and their effects on the performance of pigs under these circumstances.

An increase in glucocorticoid circulation because of chronic stress can reduce performance in pigs (Foury et al., 2005). This reduction may be related to an increase in muscle proteolysis and a reduction in protein synthesis. This mechanism is defined as muscle atrophy induced by glucocorticoids (Schakman et al., 2013). The detailed mechanism of the response to glucocorticoids during stress events has been presented by other researchers (Sapolsky et al., 2000; Bjelakovic et al., 2007; Lee et al. 2015), but simply put, the decrease in performance can be a direct effect of cellular damage due to the accumulation of reactive oxygen species (such as hydrogen peroxide and superoxides) within the cell, a condition known as oxidative stress.

In conclusion, Cr propionate did not improve the performance and carcass characteristics of pigs when subjected to chronic stress due to physical space restriction under the conditions of the current study. These results differ from other published literature which has demonstrated positive effects of Cr supplementation under stress conditions. Reducing the floor space allowance resulted in a significant reduction in growth performance, illustrating the importance of adequate floor space withing the growing and finishing stages.
